# Exploring the nucleotide molecular mechanism of compound kushen injection for lung adenocarcinoma based on network pharmacology and molecular docking

**DOI:** 10.3389/fonc.2022.1013052

**Published:** 2022-11-07

**Authors:** Zhangpeng Ye, Xin Yao, Zhubei Chen, Qin Jin, Qingsheng You

**Affiliations:** ^1^ Department of Cardiothoracic Surgery, Affiliated Hospital of Nantong University, Nantong, China; ^2^ Nantong University Medical School, Nantong, China

**Keywords:** lung adenocarcinoma, compound kushen injection, nucleotide, network pharmacology, molecular docking

## Abstract

Compound kushen injection is an effective traditional Chinese medicine for the treatment of lung cancer. However, its influence on the survival and prognosis of patients with lung adenocarcinoma patients was less studied; especially its pharmacological mechanism remains to be further elucidated. In the present study, we adopted a network pharmacology (NP)-based approach to screening effective compounds, screening and predicting target genes, analyzing biological functions and pathways, constructing a regulatory network and protein interaction network, and screening the key targets. Moreover, mass survival analysis and molecular docking were conducted. In the end, 35 key compounds and four possible central target genes were screened out, which could be used for the treatment of lung adenocarcinoma and affected the survival and prognosis of patients with lung adenocarcinoma. In addition, their key compounds had good docking affinity. Enrichment analysis showed that CKI might affect the treatment and prognosis of lung adenocarcinoma patients by regulating the PI3K–Akt signaling pathway, TNF signaling pathway, non-small cell lung cancer, Hepatitis C, etc. We discussed the pharmacological mechanisms and potential therapeutic targets of CKI in the treatment of lung adenocarcinoma, which verified the effect of CKI on the prognosis and survival of patients. The present study might promote the further clinical application of CKI and provide a theoretical basis for further experimental studies.

## Introduction

According to statistics, prostate, lung and bronchus (referred to as lung hereafter), and colorectal cancers (CRCs) accounted for 46% of all incident cases in men, with prostate cancer alone accounting for 26% of diagnoses. For women, breast cancer, lung, and CRCs accounted for 50% of all new diagnoses, with breast cancer alone accounting for 30% of female cancers (1). The most prevalent lung cancer, accounting for up to 45.5% of cases, is lung adenocarcinoma (2). The current standard of care for non-small-cell lung cancer (NSCLC) includes surgery, traditional chemotherapy and radiotherapy, targeted therapy, and immunotherapy (3). The likelihood of adverse effects may be increased by certain of these methods, such as chemotherapy administered alone (4). As a result, individuals with lung adenocarcinoma require a treatment strategy that is more sensible, affordable, and less likely to cause side effects.

In China, traditional Chinese medicine (TCM) has been used extensively in clinical settings and is becoming more and more popular worldwide (5). TCM combination therapy, particularly in the latter stages of malignant tumors, has come to be recognized as one of the essential therapies in the treatment of malignant tumors in recent years (6). Compound kushen injection is a precious compound medicine of traditional Chinese medicine with a long history and is one of the multi-target anti-tumor Chinese patent drugs (7). Modern pharmacological studies show that the main constituents of CKI are kushen and baituling (8). In addition, multiple meta-analyses have reported that the compound *Radix sophoginseng* injection combined with chemotherapy can enhance the treatment effectiveness, improve the quality of life of patients with NSCLC, and reduce the adverse events caused by chemotherapy alone (9). However, the underlying mechanism of compound kushen injection in the treatment of lung adenocarcinoma is still unclear and needs further study. With the development of the modernization of traditional Chinese medicine, network pharmacology (NP) has emerged as an advantageous method for the study of traditional Chinese medicine. It provides a new perspective for investigating TCM at the molecular level and was consistent with the natural characteristics of TCM prescription (10). Therefore, in the current study, we utilized NP to study the specific effect and potential mechanism of CKI on patients with lung adenocarcinoma.

## Materials and methods

### Construction of protein interaction network diagram and screening of core genes

To guarantee the reliability of the results, we loaded 196 genes into the STRING database platform. The PPI interaction network was further screened and integrated based on an interaction score of 0.9 and the removal of unrelated nodes (11). Next, as shown in **Table 3**, these genes were re-introduced into Cytoscape 3.8.0 and screened by DC, CC, EC, LAC, and NC with Perl software (12). The genes, which were all larger than the median value of the above conditions, were filtered out, and 44 genes were obtained in the end.

### Screening of effective components and targets of traditional Chinese medicine

According to the keywords of kushen and baituling from the database system of traditional Chinese medicine pharmacology (TCMSP, http://lsp.nwu.edu.cn/tcmsp.php), we extracted their active ingredients and target genes. The effective ingredients of the two medicines were identified according to oral bioavailability (OB) ≥30% and drug-likeness (DL) ≥0.18. OB refers to the proportion of the quantity of the drug in the preparation that is absorbed into human circulation. DL refers to the similarity of the compound to the known drug. Then we verified it by UniProt (https://www.uniprot.org/ ) (13) and the full name of the gene was converted into a symbol for subsequent analysis.

### Screening lung adenocarcinoma-related genes

Next, lung adenocarcinoma-related genes were extracted from the Online Mendelian Inheritance in Man (OMMI, http://www.ncbi.nlm.nih.gov/omim ), Pharmacogenetics and Pharmacogenomics Knowledge Base (PharmGkb, https://www.pharmgkb.org/ ), the Human Gene Database (GeneCards, http://www.genecards.org ), the Target Database (TTD, http://db.idrblab.net/ttd/ ), and Drug Bank (https://www.drugbank.ca/ ). We transfused lung adenocarcinoma to extract relevant lung adenocarcinoma genes and integrated them to serve as a basis for future analysis of common targets of drug diseases.

### Drug disease-associated gene intersection

Perl software was used to remove duplicate genes and sort out 196 intersection genes related to compound kushen injection and lung adenocarcinoma. After that, a Venn diagram was made by R software (14).

### Establishment of a Chinese medicine compound regulation network

The effective components of CKI and 196 drug disease target genes were introduced into Cytoscape3.8.0 to construct the regulatory network diagram of TCM compound.

### Construction of GO and KEGG enrichment analysis graphs

Clusterprofiler’s functional R package was utilized for GO and KEGG pathway enrichment analysis of target genes (15). GO enrichment analysis included MF (Molecular Function), BP (Biological Process), and CC (Cellular Components) analysis, and only the top 10 were shown for each. In KEGG enrichment analysis, the top 30 were screened according to p-value ≤0.05 and q-value (corrected p-value) ≤0.05. In the end, we independently selected the non-small cell lung cancer pathway (HSA05223 pathway) that might be associated with lung adenocarcinoma for analysis.

### Screening the intersection genes related to prognosis and drug regulation of lung adenocarcinoma

Clinical information on lung adenocarcinoma was extracted from The Cancer Genome Atlas (TCGA) database. We combined these genes associated with prognosis with 196 target genes using Perl software. Then we screened 38 genes with P <0.05 using R software and intersected them with the previous 44 genes. A Venn diagram was used to display the results.

### Molecular docking

We chose four genes based on the p-values of eight target genes. For molecular docking, water molecules and ligands were first removed by Pymol software, and then protein receptor structures were hydrogenated and then derived by AutoDock software. In addition, we first used PubChem (https://pubchem.ncbi.nlm.nih.gov/) to find the ligand 2D structure, used the ChemOffice software to convert it to a 3D structure, and then used the Auto Vina to calculate the minimum free energy and do molecular docking. Finally, we made the graph through Pymol software.

## Results

### Analysis of active components and target genes of CKI and target genes of lung adenocarcinoma

**Figure 1:**
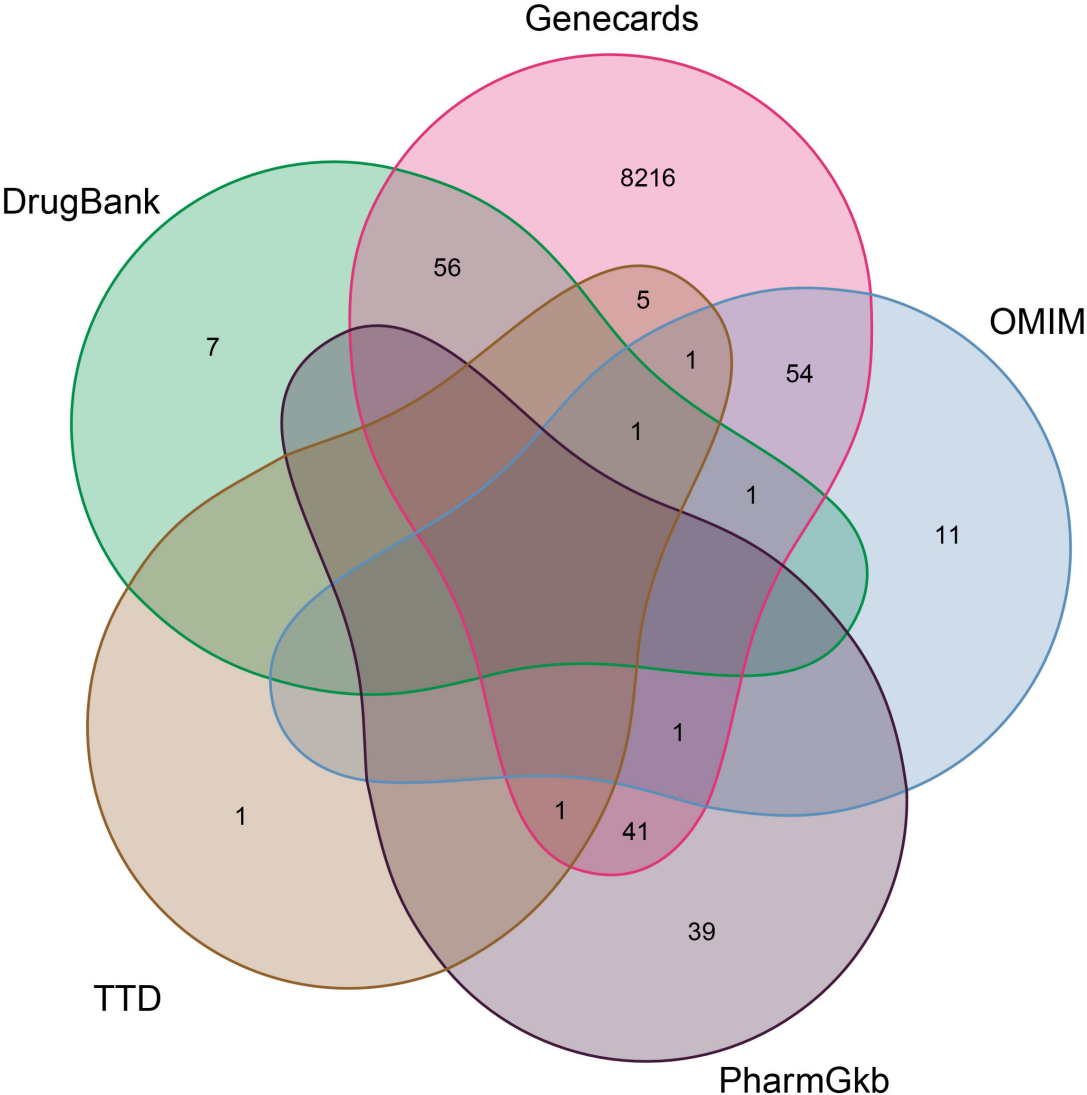
The Venn diagram of the distribution of different gene databases in lung adenocarcinoma. The number distribution of five gene data banks of LUAD and takes the union of them. The red oval represents the identified Genecards compounds. The green oval represents the identified DrugBank compounds. The purple oval represents the identified PharmGkb compounds. The gray and blue ovals represent the TTD and OMIM compounds respectively.

**Figure 2:**
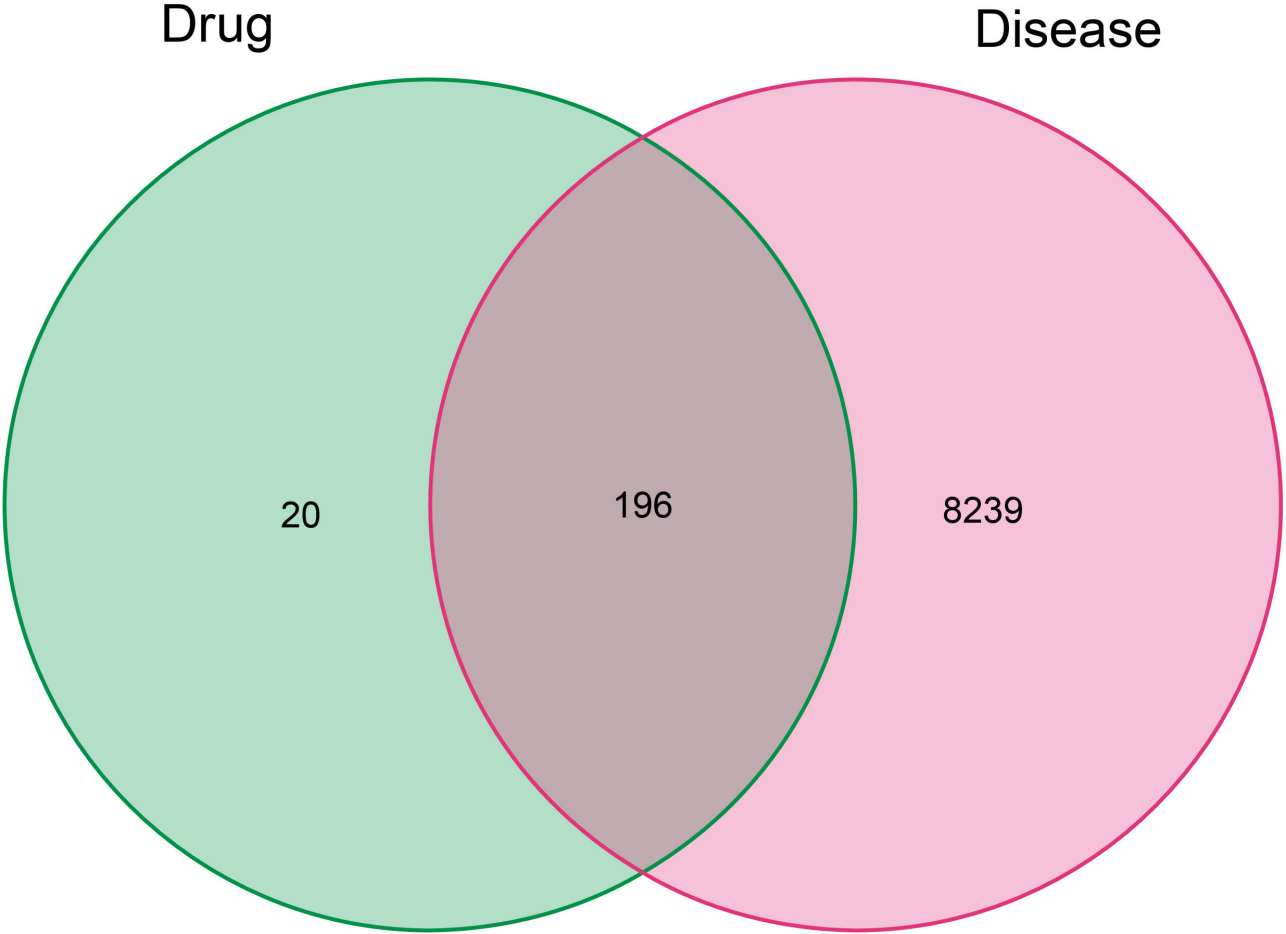
The Venn diagram of related target genes in compound kushen injection and combined lung adenocarcinoma genes. The red and green ovals represent the identified LUAD and drug targets respectively.

According to the TCMSP database, we sorted out a total of 58 active components of CKI (see **Tables 1**, **2** for details) and 216 target genes. A total of 8,435 genes in lung adenocarcinoma were collected from OMMI, PharmGkb, TTD, DrugBank, and GeneCards databases (**Figure 1**). The Venn diagram of 196 intersected genes was obtained by R software (**Figure 2**). We concluded that these intersection genes might play an important role in the treatment of lung adenocarcinoma by CKI.

**Table 1:** Core components of kushen.

MOLID	Molecule name	OB	DL
MOL001040	(2R)-5,7-dihydroxy-2-(4-hydroxyphenyl)chroman-4-one	42.36	0.21
MOL001484	Inermine	75.18	0.54
MOL003542	8-Isopentenyl-kaempferol	38.04	0.39
MOL003627	sophocarpine	64.26	0.25
MOL003648	Inermin	65.83	0.54
MOL003673	Wighteone	42.8	0.36
MOL003676	Sophoramine	42.16	0.25
MOL003680	sophoridine	60.07	0.25
MOL000392	formononetin	69.67	0.21
MOL004580	cis-Dihydroquercetin	66.44	0.27
MOL004941	(2R)-7-hydroxy-2-(4-hydroxyphenyl)chroman-4-one	71.12	0.18
MOL005100	5,7-dihydroxy-2-(3-hydroxy-4-methoxyphenyl)chroman-4-one	47.74	0.27
MOL005944	matrine	63.77	0.25
MOL000006	luteolin	36.16	0.25
MOL006561	(+)-14alpha-hydroxymatrine	35.73	0.29
MOL006563	(+)-9alpha-hydroxymatrine	32.04	0.29
MOL006564	(+)-allomatrine	58.87	0.25
MOL006565	AIDS211310	68.68	0.25
MOL006566	(+)-lehmannine	58.34	0.25
MOL006568	isosophocarpine	61.57	0.25
MOL006569	(-)-14beta-hydroxymatrine	37.26	0.29
MOL006570	(-)-9alpha-hydroxysophoramine	35.23	0.29
MOL006571	anagyrine	62.01	0.24
MOL006572	1,4-diazaindan-type,alkaloid,flavascensine	34.64	0.24
MOL006573	13,14-dehydrosophoridine	65.34	0.25
MOL006582	5伪,9伪-dihydroxymatrine	40.93	0.32
MOL006583	7,11-dehydromatrine	44.43	0.25
MOL006596	Glyceollin	97.27	0.76
MOL003347	hyperforin	44.03	0.6
MOL006613	kushenin	47.62	0.38
MOL006619	kushenol J	51.39	0.74
MOL006620	kushenol J_qt	50.86	0.24
MOL006622	kushenol O	42.41	0.76
MOL006623	kushenol,t	51.28	0.64
MOL006626	leachianone,g	60.97	0.4
MOL006627	Lehmanine	62.23	0.25
MOL006628	(+)-Lupanine	52.71	0.24
MOL006630	Norartocarpetin	54.93	0.24
MOL000456	Phaseolin	78.2	0.73
MOL006649	sophranol	55.42	0.28
MOL006650	(-)-Maackiain-3-O-glucosyl-6'-O-malonate	48.69	0.52
MOL006652	trifolrhizin	48.53	0.74
MOL000098	quercetin	46.43	0.28

**Table 2:** Core components of baituling.

MOLID	Molecule name	OB	DL
MOL013117	4,7-Dihydroxy-5-methoxyl-6-methyl-8-formyl-flavan	37.03	0.28
MOL013118	Neoastilbin	40.54	0.74
MOL013119	Enhydrin	40.56	0.74
MOL013129	(2R,3R)-2-(3,5-dihydroxyphenyl)-3,5,7-trihydroxychroman-4-one	63.17	0.27
MOL001736	taxifolin	60.51	0.27
MOL000358	beta-sitosterol	36.91	0.75
MOL000359	sitosterol	36.91	0.75
MOL004328	naringenin	59.29	0.21
MOL000449	Stigmasterol	43.83	0.76
MOL004567	isoengelitin	34.65	0.7
MOL004575	astilbin	36.46	0.74
MOL004576	taxifolin	57.84	0.27
MOL004580	cis-Dihydroquercetin	66.44	0.27
MOL000546	diosgenin	80.88	0.81
MOL000098	quercetin	46.43	0.28

### Functional and pathway enrichment analysis of intersecting genes

**Figure 3:**
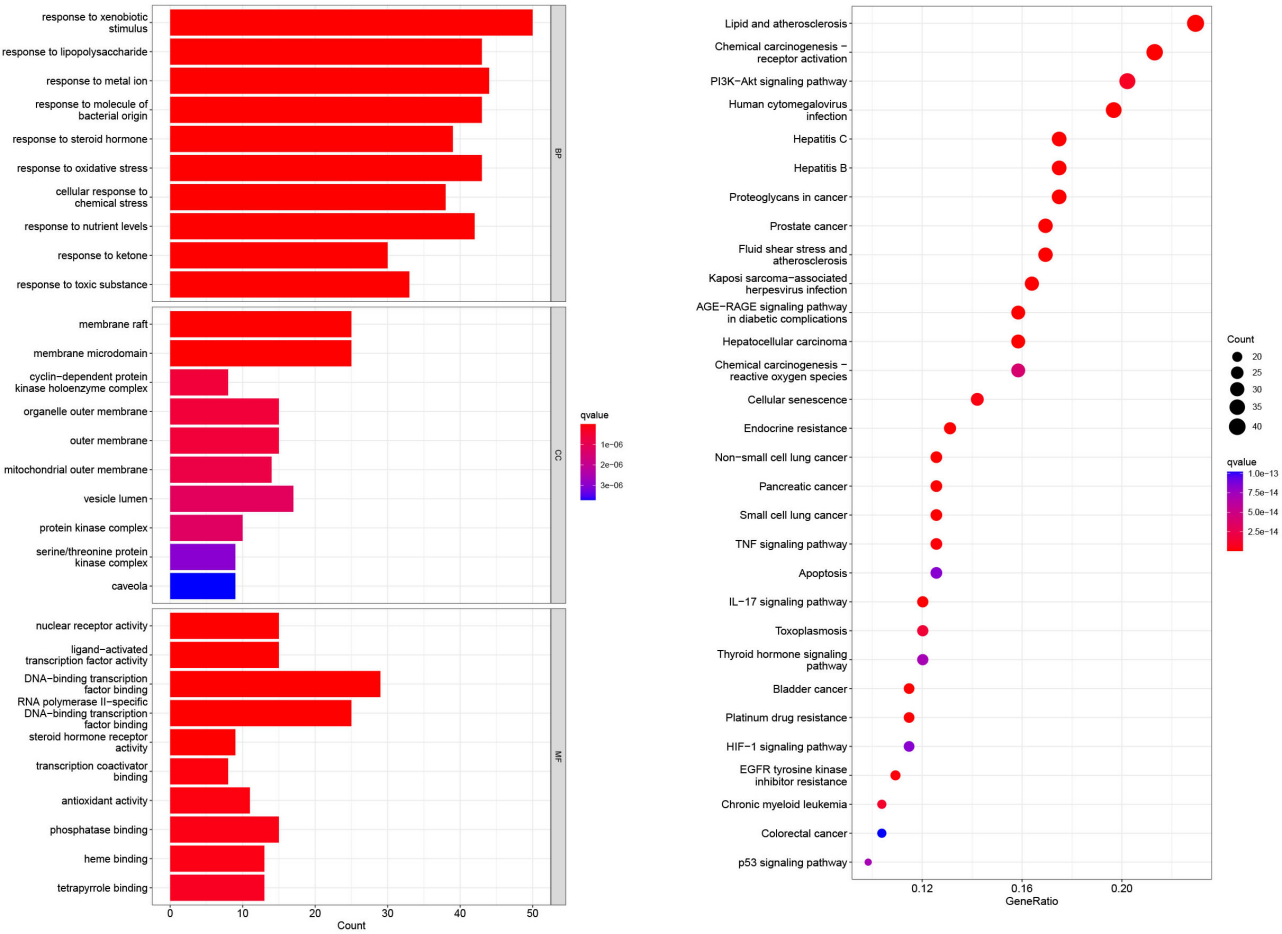
The GO enrichment analysis of core nodes. Including cellular components, molecular functions, and biological processes. The top 30 pathways for KEGG enrichment analysis of compound kushen injection.

**Figure 4:**
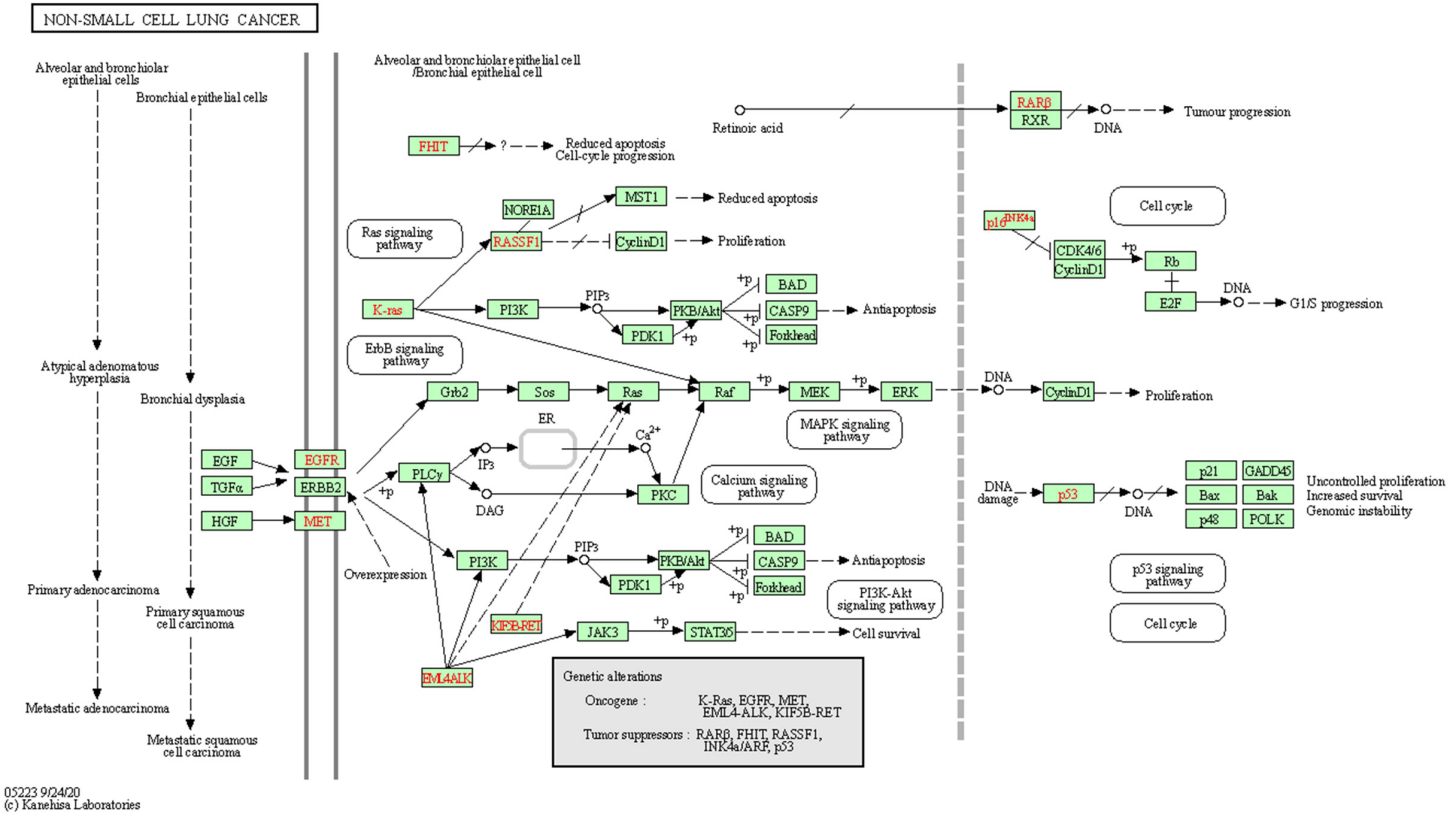
Map of gene pathways associated with non-small cell lung cancer. The red rectangular nodes represent the associated genes in 196 drug-disease overlapping genes.

GO and KEGG enrichment analyses were performed on the 196 common target genes of diseases and drugs. According to the filtering criteria, P ≤0.05 and Q value ≤0.05, the top 10 functions in GO enrichment analysis were discovered. As shown in **Figure 3**, the top 10 biological processes were related to xenobiotic stimulation, lipopolysaccharide, nutrient levels, toxic substances, etc. For cellular components, the targets were enriched in membrane raft, mitochondrial outer membrane, cyclin-dependent protein kinase holoenzyme complex, oxidative stress, etc. Molecular function analysis included nuclear receptor activity, ligand-activated transcription factor activity, DNA-binding transcription activity factor binding, RNA polymerase II-specific DNA-binding transcription factor binding, etc. In the KEGG enrichment analysis, it screened the top 30 pathways related to non-small cell lung cancer, small cell lung cancer, lipid and atherosclerosis, hepatitis C, chemical carcinogenesis-receptor activation, proteoglycans in cancer, chemical carcinogenesis-reactive oxygen species, PI3K-Akt signaling pathway, TNF signaling pathway, IL-17 signaling pathway, thyroid hormone signaling pathway, etc. The involvement of CKI in the therapy of lung adenocarcinoma was further confirmed by the mapping of pathways linked to non-small cell lung cancer (**Figure 4**).

### Construction of drug component-gene regulatory network

**Figure 5:**
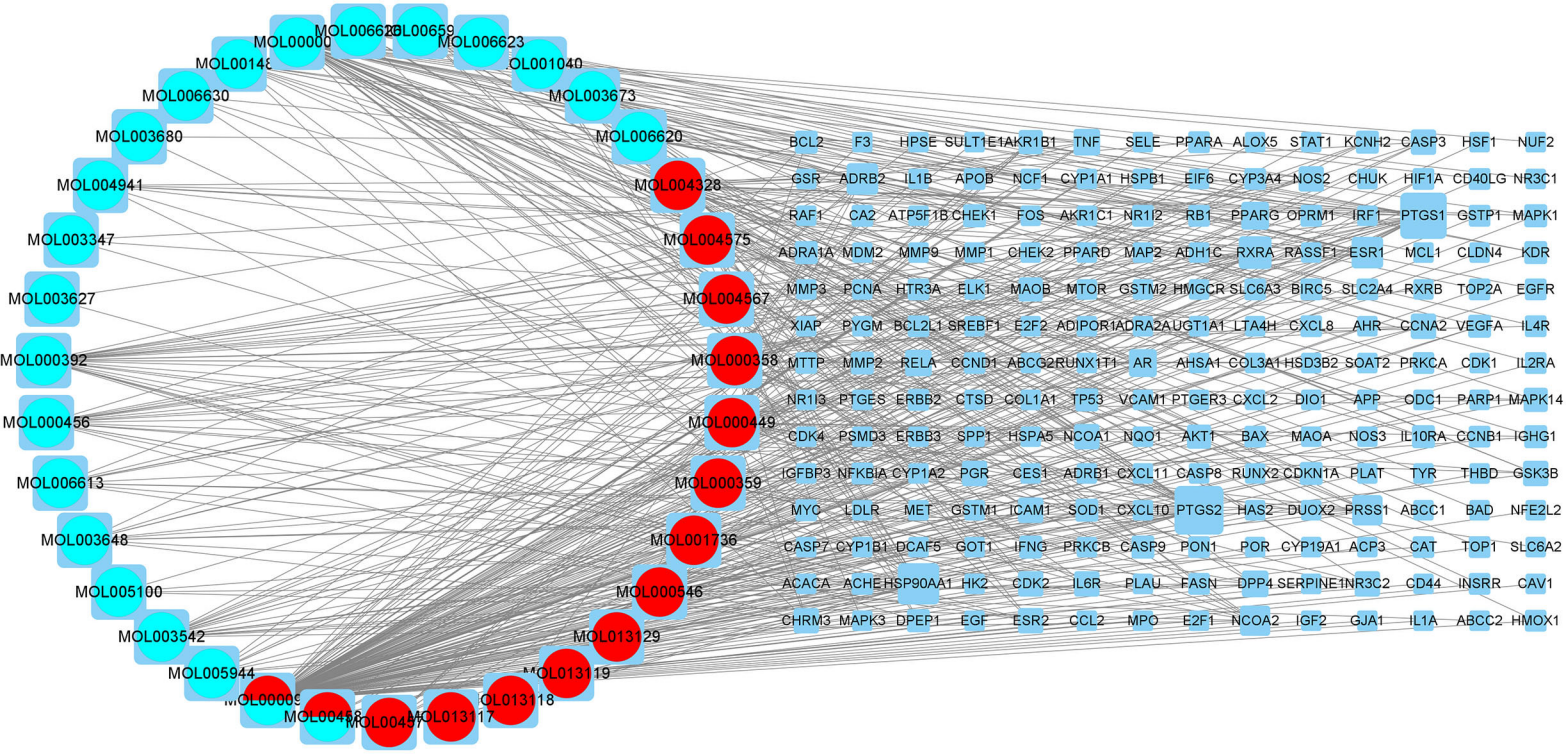
The interaction network of drugs-compounds-hub target genes-pathways. The circular nodes stand for the active ingredients of compound kushen injection and the blue square nodes stand for genes. The greater degree of the hub genes and compounds, the greater size of the nodes. The blue circular nodes represent the active ingredients of kushen and the red round nodes represent the active ingredients of baituling. The mixed colors represent ingredients containing two traditional Chinese medicines.

We loaded 35 active components and 196 lung adenocarcinoma-related genes from compound kushen injection into Cytoscape 3.8.0 and a regulatory network was also constructed (**Figure 5**). The circular nodes stood for the active ingredients of compound kushen injection and the blue square nodes stood for genes. The size of the nodes increases with the degree of hub genes and compounds. The blue circular nodes represented the active ingredients of kushen and the red round nodes represented the active ingredients of baituling. The mixed colors represent ingredients containing both of the two traditional Chinese medicines. It has been demonstrated unequivocally that a single pharmacological component may simultaneously control many genes, as well as multiple drug components working together to regulate a target.

### Protein–protein interaction network analysis

**Figure 6:**
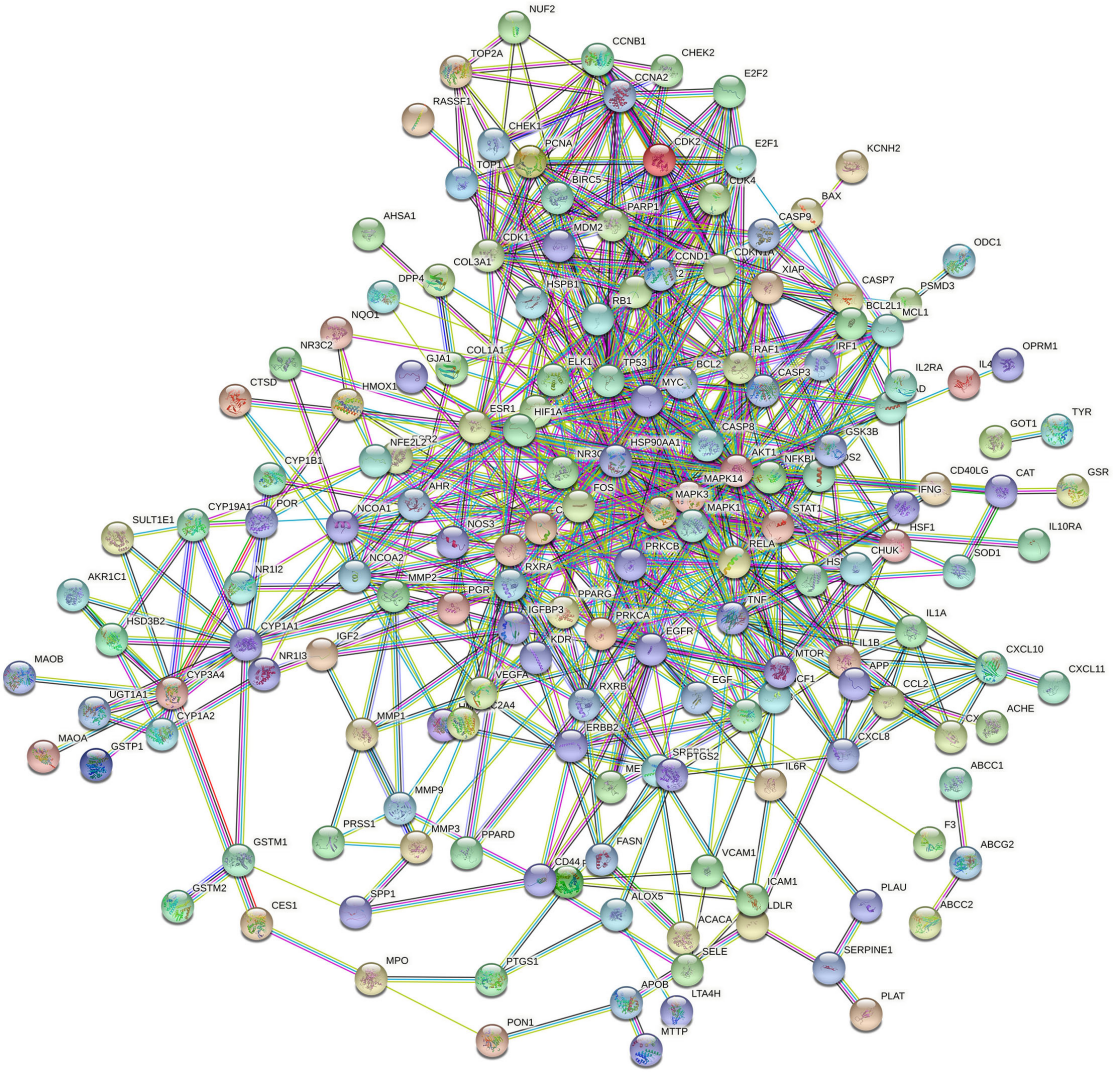
The network for 196 co-targeted genes/proteins had been selected as input for PPI analysis in STRING.

**Figure 7:**
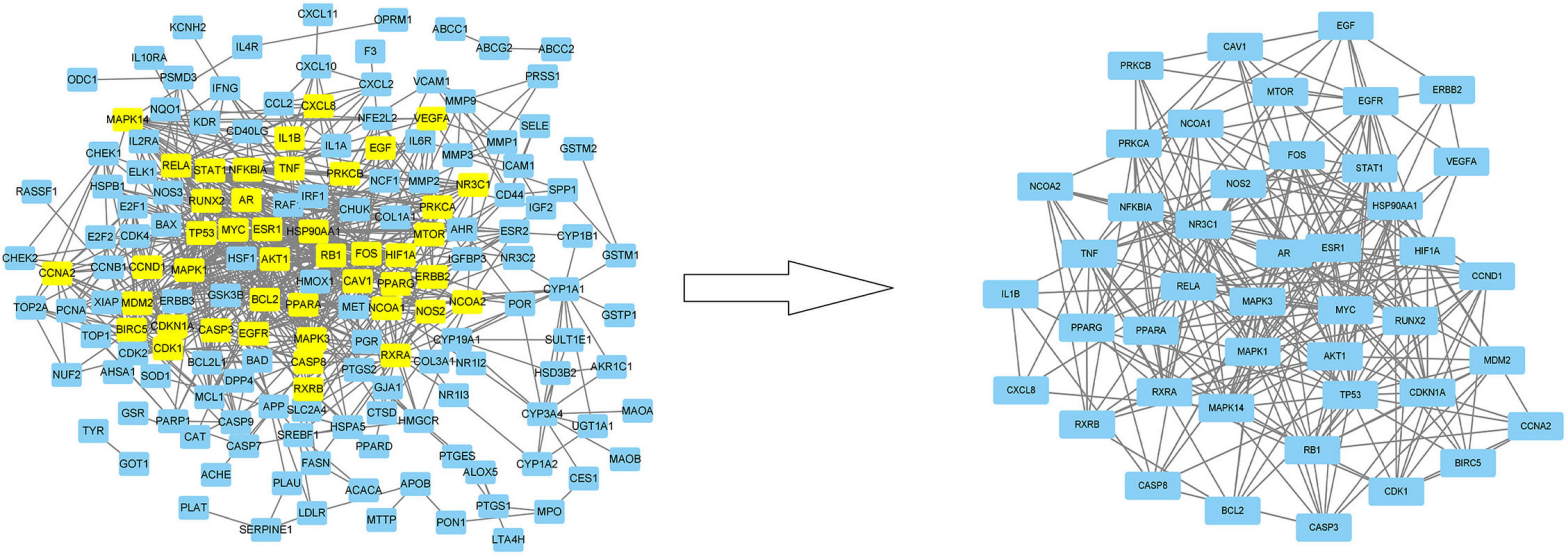
The protein-protein interaction (PPI) network. The PPI network contains 165 nodes and 729 edges. The areas highlighted in yellow are represented the candidate targets ranked in the median values for the DC, BC, CC, EC, LAC, and NC. The PPI network for the candidate targets ranked in the median values for the DC, BC, CC, EC, LAC, and NC. The PPI network contains 44 nodes and 429 edges. The areas highlighted in yellow are represented the potential targets with the median values for the DC, BC, CC, EC, LAC, and NC.

**Figure 6** showed that the network for 196 co-targeted genes had been selected as input for protein-protein interaction (PPI) analysis in STRING. **Table 3** and **Figure 7** showed the screening of genes according to the median values of DC, CC, EC, LAC, and NC, and a vivid description of the process, respectively. It was shown that 44 target genes were the main targets for CKI to participate in the regulation and treatment of lung adenocarcinoma.

**Table 3:** Information on 44 core targets.

name	Betweenness	Closeness	Degree	Eigenvector	LAC	Network
RXRA	2687.96	0.13922	25	0.12621	4.88	12.3559
MAPK14	1320.3	0.13782	26	0.17326	6.30769	11.1306
CDKN1A	233.359	0.13454	21	0.15742	9.52381	13.2798
AKT1	1869.26	0.14017	38	0.22659	7	19.8584
BCL2	107.624	0.13344	11	0.09191	5.63636	6.86429
IL1B	195.7	0.12934	11	0.04401	4.90909	7.05476
RXRB	250.921	0.13301	12	0.07465	4	5.89621
HSP90AA1	2771.82	0.14114	43	0.23762	6.93023	22.243
PRKCB	85.6513	0.13089	11	0.07095	3.09091	3.80556
RUNX2	348.262	0.13355	17	0.1293	5.05882	5.93452
ESR1	1692.79	0.14005	29	0.19676	7.31034	14.5341
CXCL8	104.972	0.12813	8	0.03548	4.5	5.2
STAT1	1018.84	0.13805	22	0.14938	5.45455	8.69562
MAPK1	1229.31	0.14089	36	0.23698	8.16667	19.4127
NCOA1	585.12	0.1329	17	0.07494	3.52941	6.92806
RELA	1631.23	0.13969	31	0.18097	6.96774	16.1015
PPARA	728.316	0.13689	15	0.11076	5.73333	6.98726
NCOA2	168.545	0.13078	12	0.05747	4	5.76299
ERBB2	152.56	0.13026	9	0.0467	2.88889	3.64286
MTOR	260.533	0.13388	11	0.07371	3.45455	4.01944
AR	256.025	0.13644	15	0.10626	3.46667	4.0092
CAV1	810.179	0.13576	17	0.08522	2.70588	4.175
EGFR	1451.62	0.13758	22	0.13507	5.72727	9.89161
NFKBIA	243.095	0.13599	15	0.11526	6.4	7.94185
NOS2	227.327	0.13498	12	0.0959	5.16667	5.91515
NR3C1	198.853	0.13701	16	0.13489	6.125	7.04221
CDK1	514.874	0.13476	18	0.11604	8.11111	11.7764
CCND1	308.934	0.13421	20	0.14082	7.9	11.2294
PPARG	267.442	0.13509	12	0.07464	2.83333	3.62045
CASP8	609.654	0.13377	15	0.08938	3.33333	5.52082
CASP3	710.58	0.13454	19	0.12226	5.78947	9.58247
VEGFA	493.724	0.13047	12	0.05159	2.83333	4.9013
PRKCA	711.546	0.13667	17	0.1049	4.35294	5.95765
FOS	1103.71	0.13816	24	0.15232	6.16667	10.0468
CCNA2	98.4386	0.12853	15	0.08513	7.73333	10.7219
EGF	364.006	0.13226	11	0.06158	3.63636	4.375
TP53	2872.22	0.14101	40	0.25484	8.5	21.4317
RB1	479.563	0.13712	23	0.17401	8.43478	12.4595
BIRC5	230.993	0.13037	12	0.07336	3.83333	5.2803
MAPK3	1344.82	0.1415	38	0.25227	8.89474	21.9116
MDM2	471.96	0.1341	17	0.12263	6.11765	7.78631
MYC	436.551	0.13851	23	0.19177	8.78261	11.7684
HIF1A	269.255	0.13793	17	0.15341	7.41176	8.39529
TNF	1023.1	0.1361	23	0.11493	5.73913	11.9109

### Results of the intersection of prognostic genes and drug regulation genes in lung adenocarcinoma

**Figure 8:**
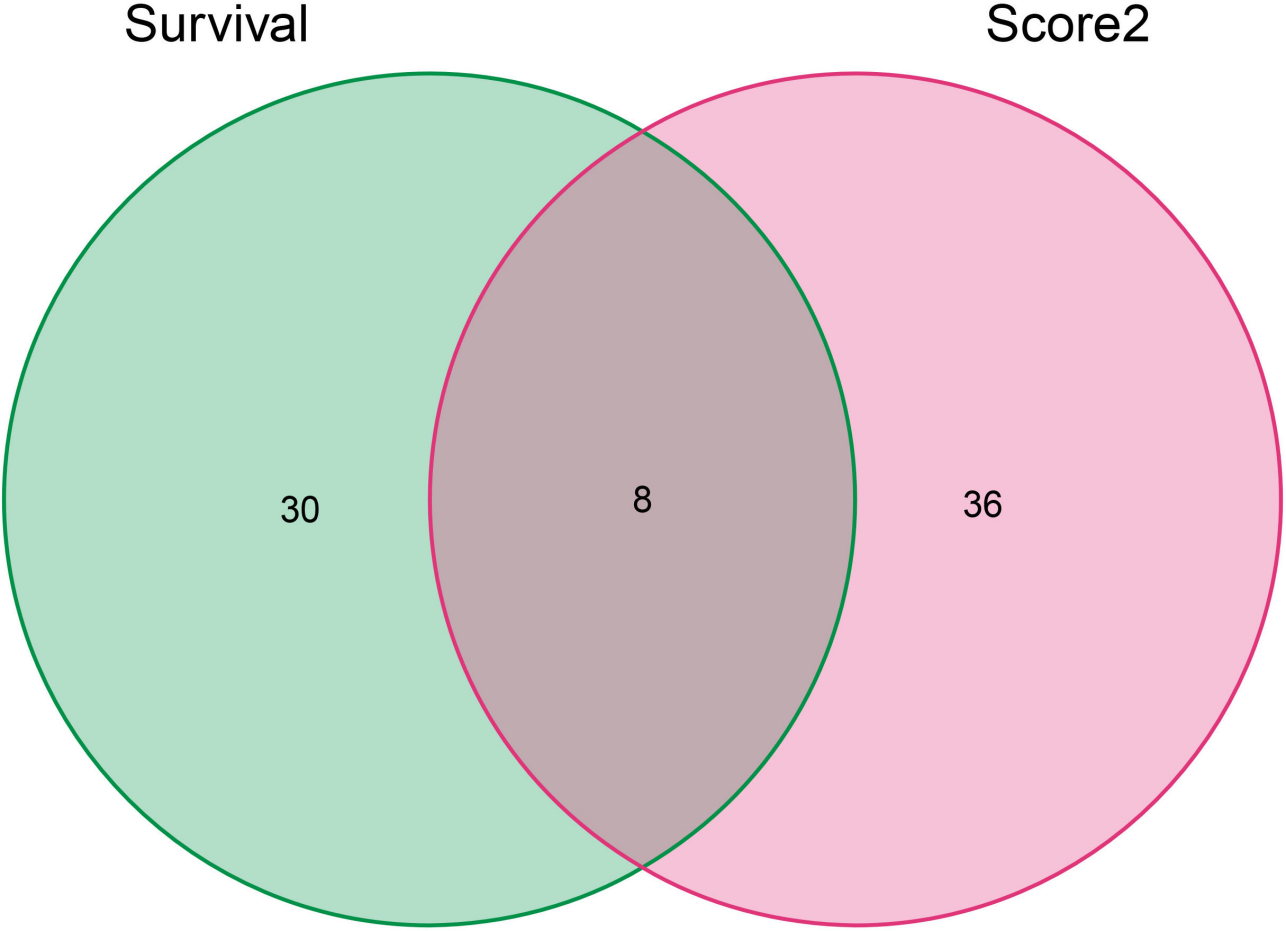
The Venn diagram of genes screened from the PPI network above and genes associated with a survival prognosis of lung adenocarcinoma. The green and red ovals represent the identified prognostic gene and PPI network gene respectively.

**Figure 9:**
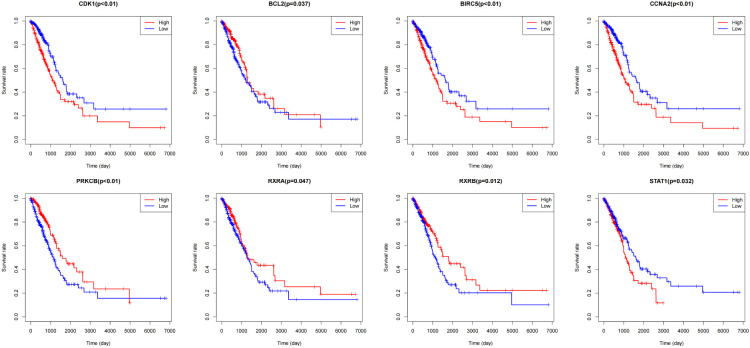
Survival curves of BCL2, BIRC5, CCNA2, CDK1, PRKCB, RXRA, RXRB, and STAT1 in lung adenocarcinoma patients.

The Cancer Genome Atlas (TCGA) is a public-funded project that aims to catalog and discover major cancer-causing genomic alterations to create a comprehensive “atlas” of cancer genomic profiles (16). The 196 drug-disease genes were collated by Perl software using clinical data obtained from the TCGA. According to P <0.05, eight intersection genes were filtered out by prognostic genes and drug regulatory genes, which were shown in **Figure 8**, namely BCL2, BIRC5, CCNA2, CDK1, PRKCB, RXRA, RXRB, and STAT1. In addition, batch survival analysis was performed in **Figure 9**, which indicated that CKI could improve the survival and prognosis of patients by regulating these genes.

### Analysis of molecular docking

**Figure 10:**
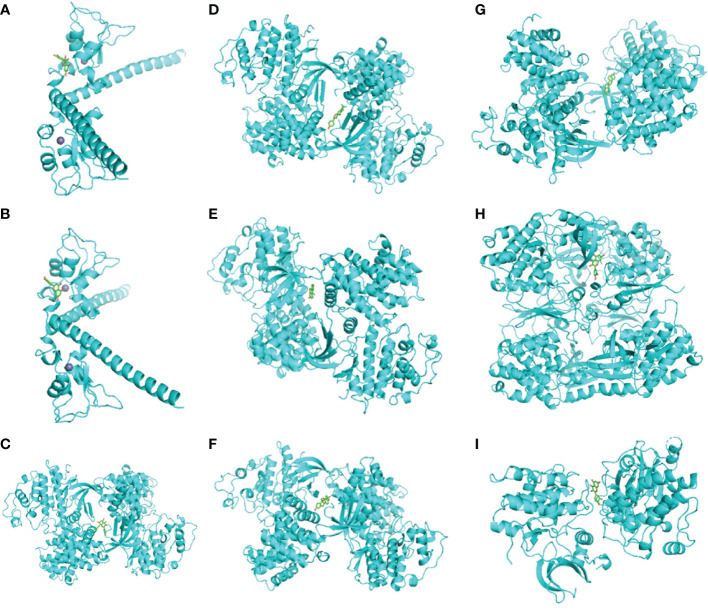
**(A)** BIRC5-quercetin, **(B)** BIRC5-luteolin, **(C)** CCNA2-8_Isopentenyl_kaempferol, **(D)** CCNA2-Wighteone, **(E)** CCNA2-formononetin, **(F)** CCNA2-Glyceollin, **(G)** CCNA2-Phaseolin, **(H)** CDK1-quercetin, and **(I)** PRKCB-quercetin.

Eight genes screened from the PPI network were further selected based on p <0.01. In the end, four genes were obtained, and their protein receptors and the corresponding ligand molecules were identified. They were BIRC5 (PDB ID 3UEG), CCNA2 (PDB ID 1H1P), CDK1 (PDB ID 6GU7), and PRKCB (PDB ID 2I0E). **Table 4** showed that we used AutoDock Vina to butt each core target and its corresponding effective distributor, and the minimum free energy was calculated. The stability of the two increases with decreasing free energy (17). Finally, Pymol software is used to produce the diagram (**Figure 10**). This finding suggests that their combination might be crucial in the treatment of lung cancer in humans using compound kushen injection.

**Table 4:** Docking scores of the active ingredients of compound kushen injection with their potential targets.

Targets	Compounds	Docking affinity score (Kcal/mol)
BIRC5	luteolin	-8.2
BIRC5	quercetin	-7.7
CCNA2	8-Isopentenyl-kaempferol	-7.8
CCNA2	formononetin	-7.3
CCNA2	Glyceollin	-8.2
CCNA2	Phaseolin	-8.2
CCNA2	Wighteone	-7.5
CDK1	quercetin	-7.4
PRKCB	quercetin	-7.5

## Discussion

Lung adenocarcinoma is the most common type of lung cancer and one of the malignancies with a high fatality rate worldwide (18). CKI showed effectiveness when combined with chemotherapy drugs to treat non-small cell lung cancer (19). In this study, we screened the genes of drug active ingredients and drug-disease intersection. Next, we did GO and KEGG enrichment analysis, construction of TCM compound–target pathway network, LUAD target protein interaction network analysis, screening of PPI network core, prognostic survival analysis, and molecular docking method analysis. All of the aforementioned studies have indicated the mechanism by which CKI affects lung cancer treatment, prognosis, and survival.

We discovered through GO and KEGG enrichment analysis that the effective targets of CKI were related to responses to oxidative stress, nutritional levels, and toxic chemical in mitochondria, among other things. Response to oxidative stress could be used to study the treatment of lung adenocarcinoma, for example, KEAP1/NRF2 mutation was a major gene subtype of lung adenocarcinoma, which activated endogenous oxidative stress response and underwent significant metabolic recombination to support enhanced antioxidant generation to treat cancer (20). Response to a toxic substance can also be used in the treatment of lung adenocarcinoma (21). According to studies, the medications work to treat lung adenocarcinoma by reducing the permeability of the mitochondrial membrane (22). We also found the PI3K-Akt signaling pathway, TNF signaling pathway, non-small cell lung cancer pathway, hepatitis C pathway, etc., and TNF signaling pathway genes were associated with the survival of lung adenocarcinoma (23). Additionally, it has been demonstrated that IFN− inhibits lung cancer by causing the PI3K-Akt pathway to become active in lung adenocarcinoma cells (24). Non-small cell lung cancer, small cell lung cancer, and hepatitis C have all been shown to play an important role in lung adenocarcinoma and prognosis prediction (25).

Subsequently, we evaluated eight targets, including BCL2, BIRC5, CCNA2, CDK1, PRKCB, RXRA, RXRB, and STAT1, based on the traditional Chinese medicine drug target pathway network, LUAD target protein interaction network, and PPI network core. In addition, we concluded that mir-15b enhanced the proliferation and migration of lung adenocarcinoma by targeting BCL2 (26) and that increased BIRC5 expression could improve the efficacy of radiotherapy for LUAD cells (27). Besides, CCNA2 is a Tanshinone IIA therapeutic target for inhibiting lung adenocarcinoma (28). Studies have proved that CDK1 is a poor prognostic biomarker of LUAD and that increased expression of CDK1 may lead to a high risk of cancer recurrence and a poor prognosis in patients with LUAD (29). Moreover, PRCKB expression was also identified as significantly associated with the survival rate of lung adenocarcinoma (30). In patients with lung adenocarcinoma, the expression of Oct4 is related to the expression of STAT1, and Oct4 binds directly to the STAT1 promoter to trans-activate STAT1 in lung adenocarcinoma cells (31). In conclusion, CKI is implicated in the occurrence, development, and prognosis of lung adenocarcinoma through several targets and multiple pathways, and it has also been confirmed that CKI has the properties of multi-component, multi-target, and multi-channel therapy. The minimum free energy obtained by molecular docking analysis also verified the rationality of the active ingredients screened by the network pharmacology method, and the 3D map vividly showed the interaction between them and predicted the interaction between ligand and target at the molecular level (32).

In summary, this study integrated information from all databases and adopted the network pharmacology method to explain the potential pharmacological mechanism of CKI therapy for lung adenocarcinoma, which involved a variety of compound targets, biological processes, molecular functions, and signaling pathways, and was also verified by molecular docking. The current study provides a method and theoretical basis for further study on the mechanisms of CKI and other anticancer agents in traditional Chinese medicine in lung adenocarcinoma. For further perspective, it will promote the development of traditional Chinese medicine in the combined treatment of cancer. While the majority of our findings were derived from computational analysis and public databases, additional experiments will be required to confirm these findings. Similar studies in recent years have analyzed the mechanisms by which CKI acts on lung cancer (33). Most research showed that a correlation study of CKI proved its effectiveness in the treatment of lung cancer, and similarly, we also investigated the pharmacological mechanisms of CKI. Furthermore, our study has supplemented the lack of further focus on specific treatment of lung cancer typing and whether the application of CKI and other traditional Chinese medicine in lung adenocarcinoma has a more significant effect on all lung cancers in terms of specific classification needs further analysis and comparison. In this study, these targeted genes were associated with prognosis analysis in specific lung adenocarcinoma patients, which laid the foundation for our subsequent comparative analysis.

## Data availability statement

The original contributions presented in the study are included in the article/**Supplementary Material**. Further inquiries can be directed to the corresponding author.

## Author contributions

ZY wrote the manuscript. JQ and ZC collected the data. ZY and XY performed the data analyses. QY and ZC directed the research and proposed changes to the manuscript, and all authors reviewed the manuscript. All authors contributed to the article and approved the submitted version.

## Acknowledgments

We sincerely thank Dr. JaMin Chen from the Affiliated Hospital of Nantong University, Jiangsu, China, for her advice and support.

## Conflict of interest

The authors declare that the research was conducted in the absence of any commercial or financial relationships that could be construed as a potential conflict of interest.

## Publisher’s note

All claims expressed in this article are solely those of the authors and do not necessarily represent those of their affiliated organizations, or those of the publisher, the editors and the reviewers. Any product that may be evaluated in this article, or claim that may be made by its manufacturer, is not guaranteed or endorsed by the publisher.
